# The acute and chronic effects of local aerobic exercise on cardiovascular physiology

**DOI:** 10.1113/EP093610

**Published:** 2026-07-13

**Authors:** Myrthe P. F. van de Ven, Janneke Vloet, Daphne Merkus, Dick H. J. Thijssen

**Affiliations:** ^1^ Department of Medical BioSciences Radboud Institute for Health Sciences Nijmegen The Netherlands; ^2^ Department of Cardiology Erasmus MC Rotterdam The Netherlands; ^3^ Institute for Surgical Research, Walter Brendel Center of Experimental Medicine University Clinic Munich, LMU Munich Munich Germany; ^4^ German Center for Cardiovascular Research (DZHK) Munich Heart Alliance (MHA), Partner Site Munich Munich Germany; ^5^ Research Institute for Sport and Exercise Sciences Liverpool John Moores University Liverpool UK

**Keywords:** blood pressure, endothelial function, haemodynamic, isometric exercise training, isometric handgrip training

## Abstract

Exercise training has beneficial effects for primary and secondary prevention of cardiovascular disease (CVD). Effects of exercise training have typically been studied in the form of whole‐body exercise training, partly driven by the assumption that larger effects of exercise training can be achieved by involving more and larger muscle groups. Nonetheless, also local aerobic exercise provides valuable insight into cardiovascular responses and the mechanisms by which exercise training leads to lower CVD risk by being able to discriminate between local and remote effects of exercise. Therefore, it is important to understand the acute effects of local exercise on cardiovascular physiology, as well as how adaptations occur following exercise training. Furthermore, acute responses to local aerobic exercise may lead to (remote) cardioprotective effects, and is associated with blood pressure‐lowering effects. Understanding these effects may help to further elucidate the mechanisms through which exercise decreases the risk of CVD, while it also highlights that local aerobic exercise may prove useful for systemic adaptations. This review aims to provide an overview of the acute and chronic effects of local aerobic exercise on cardiovascular physiology. Specifically, we describe the acute and chronic effects on local vascular haemodynamics and function, including changes in blood flow, diameter response, endothelial function and responses to ischaemia–reperfusion injury. Furthermore, the acute and chronic effects of local aerobic exercise on central haemodynamics will be discussed, including blood pressure, cardiac output and total peripheral resistance. Finally, we consider how several variables such as subject characteristics and training characteristics modulate these changes.

## Abstract (highlights)

- **What is the topic of this review?**
  The acute and chronic effects of local aerobic exercise on local vasculature and systemic haemodynamics.
- **What advances does it highlight?**
*During acute local aerobic exercise*, there is an immediate, dose‐dependent local increase in blood flow, leading to a shear stress‐mediated increase in diameter of the upstream artery. *Immediately following an acute bout of local aerobic exercise*, local endothelial function is improved, accompanied by local and remote protection against endothelial ischaemia–reperfusion injury. C*hronic local aerobic exercise* leads to local shear stress‐induced improvements in vascular structure, as well as blood pressure lowering effects.

## 1 INTRODUCTION

Physical inactivity is a leading independent predictor of disease and reduced life expectancy. It is also an important modifiable risk factor for several illnesses, including cardiovascular disease (CVD) (Al Tunaiji et al., 2019; Isath et al., 2023; Tucker et al., 2022). To reduce the burden of inactivity‐associated diseases, the WHO has provided evidence‐based recommendations to engage in physical activity. However, half of the population fails to meet these recommended physical activity levels (Guthold et al., 2018; Piercy, 2019). Together with the expected rise of CVD burden, this poses a significant challenge to the future healthcare system (McClellan et al., 2019). Understanding the benefits of exercise training is essential to optimize the timing, intensity and prescription of exercise to maximise clinical benefit. Improvements in traditional and novel risk factors (e.g., hypertension, obesity and diabetes) cannot fully explain the clinical benefits of exercise training in lowering risk for cardiovascular events (Mora et al., 2007). However, increasing evidence supports a role for cardiovascular adaptations, both in function and in structure, that contributes to the clinical effects of regular exercise training (Ho et al., 2012; Lee et al., 2024; Schroeder et al., 2019). This highlights the importance of understanding how exercise affects cardiovascular physiology.

Substantial research has focused on dynamic, whole‐body exercise training (e.g., cycling, running), partly because the popularity of this type of exercise, but also given its established relation with lowering CVD risk (Charlton & Crawford, 1997). Whole‐body exercise recruits multiple large muscle groups simultaneously and results in substantial increases in cardiac output, ventilation, sympathetic activation and systemic shear stress (Joyner & Casey, 2015). In contrast to whole‐body exercise, where multiple regulatory pathways are activated at both systemic and local level, local aerobic exercise isolates the cardiovascular response of the activated muscle only (Joyner & Casey, 2015; Molenaar et al., 2023). In this review, local aerobic exercise refers to rhythmic, submaximal activity performed by a small, isolated muscle group, typically involving a single limb or a very limited muscle mass (e.g., unilateral handgrip, knee‑extensor ergometry, or single‑leg cycling). A major advantage of examining local aerobic exercise responses is the ability to discriminate between local and remote effects of exercise. Therefore, underlying mechanisms and local effects within the working muscle can be studied in more detail, without being masked or interfered with by circulating factors induced by whole‐body exercise affecting cardiovascular health and/or systemic blood pressure changes. Furthermore, examining local exercise permits the quantification of both local and remote cardiovascular effects. Importantly, increasing evidence demonstrates the potential that, similar to whole‐body exercise, also local aerobic exercise training can achieve systemic clinical effects. For example, local aerobic exercise has blood pressure‐lowering effects that may match those of aerobic whole‐body exercise (Edwards et al., 2022). Furthermore, studies have linked local aerobic exercise to remote, cardioprotective effects (Thijssen et al., 2022). Examining local aerobic exercise, therefore, may help to further understand, but also to benefit from, the effects of exercise on cardiovascular health.

This review aims to discuss the acute effects of local aerobic exercise on central and local haemodynamics, vascular function and protection against ischaemia–reperfusion (IR) injury. Subsequently, we summarise the chronic effects of local aerobic exercise training. Finally, we discuss subject‐ and exercise‐related factors that impact the responses of local aerobic exercise on the cardiovascular physiology. Two exercise‐related factors, intensity (e.g., percentage of maximal voluntary contraction; MVC) and duration, define the ‘dose’ of exercise in this review and are used to describe the dose‐dependent effects.

## 2 WHAT ARE THE ACUTE EFFECTS OF LOCAL AEROBIC EXERCISE?

### 2.1 Local vascular haemodynamics

#### 2.1.1 Blood flow and vasodilation

Muscle blood flow is closely coupled to the metabolic demand of the tissue, which depends on the intensity of exercise and volume of muscle mass (Joyner & Casey, 2015). It is important to note that muscle contractions during local aerobic exercise mechanically affect flow, as the contracting muscles compress arterioles, capillaries and venules and consequently affect transmural pressures, thereby hampering the increase in blood flow (Charlton & Crawford, 1997; Joyner & Casey, 2015). Although blood flow is rapidly restored upon release of the muscle contraction, the duration and strength of each muscle contraction together with blood pressure determine the constantly changing pattern of blood flow in the exercising muscle (Delp & Laughlin, 1998; Joyner & Casey, 2015).

The magnitude of this so‐called exercise‐induced hyperaemia is mainly determined by vasodilation of the arterioles, which are the principal regulators of both local perfusion and total peripheral resistance (TPR) (Bagher & Segal, 2011). In a contracting muscle, there is marked vasodilation in all parts of the arteriolar tree, with the smallest arterioles being most sensitive to metabolites (VanTeeffelen & Segal, 2003, 2006). The arteriolar vasodilation produces a large decrease in local resistance and thereby enables substantial increases in muscle blood flow. To a smaller extent, enhanced microvascular perfusion also increases upstream vasodilation (Hellsten et al., 2012; Joyner & Casey, 2015; Poole & Musch, 2023).

This rapid vasodilation is a coordinated response, involving a multitude of mechanisms. First, metabolic control of the terminal arterioles plays an important role. Although the exact mechanisms are still debated, the release of adenosine by contracting skeletal muscle, as high energy stores in the muscle are depleted and/or ATP turnover is increased, is considered an important contributor (Joyner & Casey, 2015). Additional metabolic molecules implicated in rapid vasodilation include K^+^ and H^+^, released by the contracting skeletal muscle, as well as ATP and NO, released by erythrocytes (Casey et al., 2017). Subsequently, the increase in flow recruits upstream vessels through shear stress‐induced, endothelium‐mediated secretion of vasoactive substances, including NO (Green et al., 2014, 2011; Joannides et al., 1995), which is facilitated by eNOS‐Ser^1177^ phosphorylation (Casey et al., 2017) and upregulation of heat shock proteins, prostaglandins (Okahara et al., 1998), H_2_O_2_ (Liu et al., 2011) and endothelium‐derived hyperpolarizing factor (EDHF) (Busse et al., 2002). In addition, a propagated response of vasodilator signals from the microcirculation along vascular smooth muscle cells is observed (Delp & Laughlin, 1998). This further contributes to recruitment of vasodilation of the upstream resistance vessels, and seems to be evoked by acetylcholine and bradykinin in response to electrical signals between endothelial cells and vascular smooth muscle cells (Garland & Dora, 2023). Within vascular smooth muscle cells, activation of protein kinase A (PKA) and protein kinase G, inactivation of protein kinase C isoforms and activation of K^+^ channels such as K_ATP_, K_Ca_ and K_V_ channels leads to smooth muscle relaxation and vasodilation (Thijssen et al., 2022). Importantly, efforts to block single or even multiple pathways have not (markedly) changed the exercise‐induced increase in blood flow. This means that the blood flow response to exercise is incredibly robust, showing that multiple pathways or substances are working together, rather than being obligatory, to regulate the blood flow response to exercise (Joyner & Casey, 2015).

#### 2.1.2 Shear stress

As a result of (dose‐dependent) dilation of distal terminal arterioles and the subsequent increase in blood flow in proximal conduit arteries, also shear stress changes. Shear stress is defined as the frictional force exerted by flowing blood on the endothelium, which is directly related to the velocity of the blood, and inversely related to vessel diameter (Pyke & Tschakovsky, 2005). Shear stress is calculated using shear rate and blood viscosity, but blood viscosity is assumed to be unlikely to change acutely when examining local exercise in humans in vivo. Consequently, shear rate is commonly used as a surrogate for shear stress (Thijssen et al., 2019). Under resting conditions, a typical shear pattern occurs across the cardiac cycle, demonstrating antegrade shear during systole (toward the peripheral vessels) and retrograde shear during diastole (back toward the heart) (Schreuder et al., 2015).

To understand the impact of local exercise on shear stress, Cocking *et al.* examined brachial artery shear rate patterns during submaximal intermittent handgrip exercise at 25% MVC (30 min at 30 contraction–relaxation cycles/minute). The overall increase in blood flow was accompanied by an increase in antegrade shear rate, whilst retrograde shear rate became less negative (Cocking et al., 2018). Investigating a potential dose–response relation, Atkinson et al. (2015) included handgrip exercise at 5, 10 and 15% MVC (30 min at 30 contractions/min). They found the responses of mean, antegrade and retrograde shear rate during handgrip exercise to be dose‐dependent, with a higher overall flow inducing a larger increase in antegrade shear and attenuation of retrograde shear at higher intensities (Atkinson et al., 2015). They also reported that, although exercise at low intensity (5% MVC) did not alter brachial artery diameter, handgrip exercise at 15% MVC increased diameter immediately post‐exercise (Atkinson et al., 2015). To further understand blood flow responses to local handgrip exercise, Hanson & Casey (2023) investigated intermittent handgrip exercise (6 × 3 min with 20 contractions/min, 2 min rest in between) at 15% and 25% MVC as well as continuous handgrip exercise (30 min, 20 contractions/min, 25% MVC). They found that shear rate was higher at a higher intensity, for both intermittent and continuous exercise. There were no differences in retrograde shear rate between the three interventions. This suggests that, as the exercise stimulus is increased, the shear rate is also elevated in a dose‐dependent manner, while independent of the mode of exercise (Hanson & Casey, 2023). Mechanistically, shear rate causes eNOS activation (eNOS Ser^1177^ phosphorylation), triggered by mechanosensors such as cavolae and glycocalyd, and mediated by Akt, PKA, CaM kinase and/or AMP activated kinase (Erkens et al., 2017). This results in increased NO production and bioavailability (Harrison et al., 2006). This pathway may also be influenced by varying shear rate patterns (Hanson & Casey, 2023), although the impact of intermittent versus continuous shear rate patterns on eNOS activity or improved mechanosensing remains to be determined.

### 2.2 Vascular function

To examine vascular function, flow‐mediated dilation (FMD) is commonly used in human studies. This technique uses ultrasound to assess changes in conduit artery diameter in response to a brief period of ischaemia of the distal vascular bed, and has demonstrated independent prognostic value for CVD (Thijssen et al., 2019). Previous studies revealed that local aerobic exercise is capable of enhancing FMD post‐exercise, as compared to pre‐exercise. Atkinson *et al.* reported that FMD increased dose‐dependently following incremental levels of handgrip exercise at 5, 10 and 15% MVC (30 min at 30 contractions/min), with the highest intensity showing the largest increase in FMD (Atkinson et al., 2015). These observations have been confirmed by other studies, showing that 11–30 min of local handgrip exercise is sufficient to transiently improve FMD, which returns to baseline 1–2 h post‐exercise (Hanson & Casey, 2023; McPhee & Pyke, 2018; Tremblay et al., 2019). These intensity‐dependent effects on FMD may be explained by the underlying physiological stimuli. First, metabolic vasodilators such as CO_2_, adenosine and prostaglandins accumulate in proportion to the metabolic demand, enhancing vasodilation in the microvasculature (Okahara et al., 1998). Second, higher intensities of local exercise generate larger increases in antegrade shear stress (Atkinson et al., 2015). Previous work in vitro and in vivo demonstrated that such elevations in antegrade shear patterns yields an anti‐atherogenic phenotype (e.g., oscillatory shear) (Laughlin et al., 2008).

Tinken et al. (2009) further investigated the role of shear rate on the dose‐dependent change in vascular function following local aerobic exercise in humans. Participants were exposed to bilateral handgrip exercise, which acutely increased brachial artery blood flow and shear rate, but also improved FMD following 30 min of handgrip exercise (intensity matched to the mean blood flow during preceding forearm heating, 30 min at 30 contractions/min). To distinguish the effect of exercise from the increases in blood flow and shear stress, the increases in flow and shear rate during handgrip exercise were abolished by inflation of a blood pressure cuff around one forearm to sub‐diastolic pressure (60 mmHg). Abolishing the increase in flow also negated the change in FMD, suggesting that the increase in shear stress mediates the increase in FMD following a bout of acute local exercise (Tinken et al., 2009). Comparable observations were found following local heating to both forearms with a blood pressure cuff, abolishing the increase in blood flow and shear rate in the contralateral arm (Tinken et al., 2009). Since heating elevates shear without a concomitant change in metabolic components, or systemic pressure changes, this observation highlights the importance of the local increase in shear stress in mediating changes in FMD, likely due to shear stress‐induced vasodilatory effects (Niebauer & Cooke, 1996).

Next to mean shear, the shear pattern also influences the endothelial function. For example, Holder et al., performed experiments that yielded repeated exposure to increases and decreases in shear (30 min intermittent negative pressure, 10 s, −40 mmHg; 7 s, 0 mmHg), whilst mean shear across the intervention was kept near resting mean shear levels. These fluctuations in shear, without changing mean shear, yielded acute improvements in FMD (Holder et al., 2019). Also during handgrip exercises, intermittently changing shear rate patterns showed a greater impact on FMD than a continuous level of elevated shear rate (Hanson & Casey, 2023). Taken together, local aerobic exercise leads to an immediate increase in FMD following exercise, an effect that is mediated by an increase in shear stress and is likely shear pattern‐ and dose‐dependent.

Another measure of vascular function is arterial distensibility, which relates to the stiffness of arteries. This can be inferred from the pulse wave velocity (PWV), where a higher PWV indicates a lower distensibility, and thus stiffer arteries. Studies have presented conflicting results as some find local PWV to increase following isometric handgrip exercise (3 min at 30% MVC), whilst others find PWV to decrease following a combination of isometric ankle plantar‐flexion (2 min at 40% MVC) and isometric handgrip exercise (2 min at 40% MVC) (Davies et al., 2007; Reid & Conway, 2006). These conflicting results may, at least in part, be explained by differences in the balance of local and systemic effects of the local exercise stimulus, subsequently affecting PWV. For example, local aerobic exercise leads to changes in vascular smooth muscle cells in the active areas (Pilz et al., 2024), which subsequently leads to vasodilation and contributes to decreases in arterial stiffness (Bolotina et al., 1994; Davies et al., 2007; Harrison et al., 2006). On the other hand, isometric exercise at high intensity can activate the sympathetic nervous system (SNS) (Edwards et al., 2024). Activation of SNS increases systemic vasoconstrictor tone (see section 2.4), which may oppose the local vasodilator effects when evaluating the PWV (Davies et al., 2007; Edwards et al., 2024; O'Driscoll et al., 2021). This interplay between local vasodilation and systemic effects, which may be partly driven by differences in duration and intensity of the interventions, may explain the discrepancy between previous findings pertaining to the impact of local aerobic exercise on PWV. More studies are required to understand the acute effects of local aerobic exercise, and how these effects are impacted by exercise characteristics.

### 2.3 Endothelial ischaemia–reperfusion injury

IR injury refers to the phenomenon that, although re‐establishment of blood flow is essential to limit ischaemia‐related injury, reperfusion following ischaemia causes further damage to the tissue as well as the endothelial cells (Bannell et al., 2023). IR injury reflects a cascade of interrelated processes, initiated during ischaemia and amplified on reperfusion, including abrupt reactive oxygen species (ROS) overproduction leading to oxidative stress, calcium overload, endothelial dysfunction and activation of inflammatory pathways (e.g., leukocyte adhesion, complement and cytokine signalling) (Goncharov & Sharapov, 2023; Zhang et al., 2024). These events promote lipid peroxidation, protein and DNA damage and cell death (Zhang et al., 2024). This type of injury occurs following a myocardial infarction or post‐cardiac surgery, with the magnitude of IR injury being inversely related to patient outcomes (Hausenloy & Yellon, 2013). Several studies established the potential of repeated, short bouts of local ischaemia (i.e., ischaemic preconditioning; IPC) and pharmacological agents to reduce IR injury (Jones et al., 2014; Kharbanda et al., 2001; Luca et al., 2013; Michelsen et al., 2012; Seeger et al., 2015; Yamashita et al., 1999). Also exercise, especially when performed with intermittent periods of high intensity, is demonstrated to induce repeated exposure to local ischaemia (Michelsen et al., 2012; Seeger et al., 2015; Yamashita et al., 1999). Mechanistically, exercise may confer protection through activation of several potential pathways, with some also activated during IPC. One potential pathway involves shear stress‐mediated upregulation of NO production (Harrison et al., 2006), which may attenuate the ROS burst and may preserve endothelial function during perfusion (Harrison et al., 2006). Alternatively, studies found exercise to affect opioid receptors and adenosine, with studies demonstrating that these pathways have been mechanistically linked to prevention of IR injury (Miron et al., 2019; Saanijoki et al., 2018). Furthermore, exercise is known to upregulate various other signalling pathways which have also been associated with minimising IR injury (e.g., nuclear factor‐κB (NF‐κB), angiotensin II type 1 receptor, superoxide and multiple inflammatory cytokines) (Harrison et al., 2006; Zhang et al., 2024). Future studies are required to understand how and which pathways are relevant in mediating the effects of exercise related to IR injury.

Recent studies have also explored whether local aerobic exercise induces protection against IR injury. Bannell et al. (2023) examined the brachial artery endothelial IR injury in 15 young, healthy males and examined the potential protective effect of IPC and handgrip exercise (4 × 5 min with 90 s rest in between, at 50% MVC, 60 reps/min). IR injury was induced by a period of 15 min ischaemia and 15 min reperfusion. FMD was examined before and after IR, and the attenuation of FMD typically observed after IR is referred to endothelial IR injury. Both handgrip exercise and IPC showed a directionally similar effect in local and remote protection against endothelial IR injury in the contralateral arm, although the effect for handgrip exercise failed to reach statistical significance (Bannell et al., 2023). Somani et al. (2024) further explored the protective potential of local aerobic exercise in older individuals with an increased risk for CVD, and showed that a single session of handgrip (50% MVC, 25 reps/min for 4 × 5 min, 5 min rest in between) or squat exercises (15 squats/min for 4 × 5 min, 5 min rest in between) offered remote protection against endothelial IR injury in the contra‐lateral limb. This remote protection remained present for 18–24 h following 1 week of daily handgrip or squat exercises (Somani et al., 2024). Further studies are warranted to understand the potential of local exercise to protect against endothelial IR injury, but also to examine potential differences in protection in relation to group characteristics (e.g., age, CVD risk).

### 2.4 Central haemodynamics

During a bout of local aerobic exercise, central haemodynamic changes also occur, which seem to be primarily driven through activation of the SNS (Edwards et al., 2024; O'Driscoll et al., 2021). Not surprisingly, changes in these central haemodynamics are less pronounced compared to whole‐body exercise, but do demonstrate a dose‐dependent relation. When only small muscle volumes are recruited, such as the upper limbs, heart rate and stroke volume and thus cardiac output are only minimally increased compared to exercise involving a larger muscle mass (Swift et al., 2022). At higher intensity or larger muscle volumes, SNS and metaboreceptors are activated in a dose‐dependent manner, which contributes to an increase in mean blood pressure up to 35 mmHg during local exercise of the lower limbs (both studies employing 4 × 2 min wall squats, 2 min rest in between) (O'Driscoll et al., 2021; Taylor et al., 2017). The elevation in blood pressure is mainly caused by an increase in cardiac output (∼2–3 L/min), primarily mediated by an increase in heart rate (up to 40–50 bpm), while stroke volume does not change markedly or shows a small decrease (up to 20 mL) (Edwards et al., 2024; O'Driscoll et al., 2021; Taylor et al., 2017). Furthermore, the activation of SNS at the start of exercise also increases TPR, which later drops to or below pre‐exercise levels when local aerobic exercise is continued (O'Driscoll et al., 2021; Taylor et al., 2017). The reduction in TPR during successive intervals of local aerobic exercise may be the consequence of vasodilation of the arterioles (see section 2.1.1), which occurs in response to meet the high metabolic demand of the activated muscles (Taylor et al., 2017). Thus, these studies indicate that cardiac output and blood pressure are affected by local aerobic exercise, but their effects strongly depend on the intensity, volume and duration of the contractions (Edwards et al., 2024).

**FIGURE 1:**
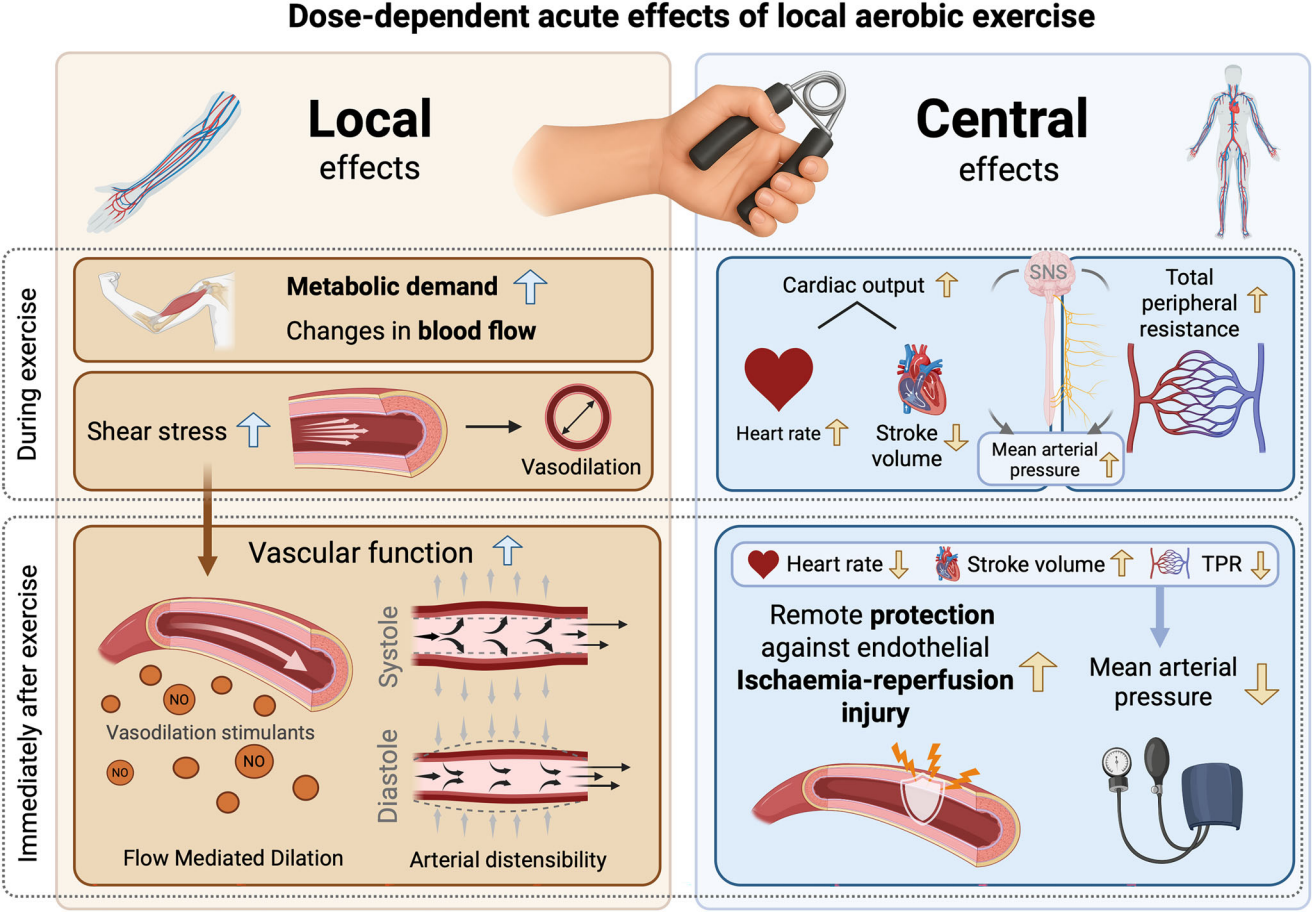
Dose‐dependent acute effects of local aerobic exercise (e.g., handgrip training), divided into local and central effects. Additionally, effects occurring during exercise are separated from effects occurring immediately after exercise. Vasodilation stimulants refer to nitric oxide (NO), adenosine and prostaglandins. TPR, total peripheral resistance.

Upon cessation of the exercise, a reduction in heart rate occurs due to parasympathetic reactivation, leading to reduced cardiac output (O'Driscoll et al., 2021; Taylor et al., 2017). Systemic TPR is demonstrated to remain low or even decrease further than during exercise (O'Driscoll et al., 2021; Taylor et al., 2017). Especially the decrease in TPR following exercise seems to contribute to a reduction in mean arterial blood pressure. In fact, blood pressure even drops below baseline levels in the initial period following exercise, which is commonly referred to as postexercise hypotension (O'Driscoll et al., 2021; Taylor et al., 2017). Whether parasympathetic activation and/or post‐exercise hypotension contribute to the chronic vascular adaptations has not been investigated to date. Figure 1 provides an overview of the acute local and central effects of local aerobic exercise.

## 3 WHAT ARE THE CHRONIC EFFECTS OF LOCAL AEROBIC EXERCISE?

### 3.1 Vascular adaptations

Several studies have examined whether local aerobic exercise training, that is, multiple days or weeks of repeated exercise interventions, leads to vascular adaptations. Previous work in normotensive individuals with a priori preserved endothelial function revealed that vascular adaptations follow a biphasic pattern in response to local aerobic exercise training. During the first weeks of training, vascular function improves. This initial improvement is later followed by structural vascular remodelling, after which shear stress normalized and function returned to baseline (Edwards et al., 2024). Indeed, Tinken et al. (2010) examined the effects of handgrip exercise for 8 weeks with increasing exercise intensity (30% MVC for 4 weeks, 40% MVC for 2 weeks, 50% MVC for 2 weeks, all 30 min at 30 contractions/min for 4 days per week). They reported initial adaptation in brachial artery vascular function (increase in FMD in weeks 2–6, which returned to baseline at week 8) and a progressive change in structure (resistance artery remodelling, evidenced by an increase in brachial artery peak reactive hyperaemia). Furthermore, progressive structural increases in femoral artery diameter and resting femoral artery blood flow were found following 8 weeks of isometric leg exercises (14% MVC, 4 × 2 min with 2 min rest, for 3 days/week) (Baross et al., 2012). To extend these observations, an improvement in arterial stiffness was seen following 4 weeks of wall squats (4 × 2 min with 2 min rest, 3 days/week) and 8 weeks of isometric handgrip training (30% MVC, 4 × 2 min with 1 min rest, 5 days/week) (Edwards et al., 2023; Okamoto et al., 2020). Together, these observations strongly support the presence of time‐dependent changes in vascular function and structure following local aerobic exercise training in normotensive individuals.

To better understand the role of the shear rate as the driving force to mediate improvements in vascular function and structure, Tinken et al. (2010) investigated adaptation in the brachial artery following 8 weeks of bilateral handgrip exercise training (30 min, 30 contractions/min). Additionally, exercise‐induced elevations in shear were unilaterally abolished using the inflation of a blood pressure cuff to sub‐diastolic levels of 60 mmHg. They reported that no changes in vascular function or structure were observed in the contralateral ‘clamped’ arm, suggesting a key role for shear stress to mediate vascular adaptations. Subsequently, Naylor et al. (2011) adopted a comparable methodological set‐up, but replaced exercise with local heating (30 min) to elevate shear stress. They confirmed that local elevations in shear stress are responsible for the local, time‐dependent adaptations in vascular function and structure. To further explore the role of shear stress, Thijssen et al. (2011) adopted the same approach and examined whether the decrease in arterial wall thickness following 8 weeks of handgrip training (30% MVC for 4 weeks, 40% MVC for 2 weeks, 50% MVC for 2 weeks, all at 30 contractions/min for 30 min, 4 days per week) is mediated through shear stress. In contrast to the observations for vascular function and structure, a comparable decline in wall thickness was found in both arms. This suggests that the change in wall thickness following exercise training is unlikely modulated by shear stress.

Mechanisms underlying the adaptation in vascular function and structure following chronic local aerobic exercise have not been investigated extensively. Based on studies pertaining to whole‐body exercise, vascular adaptations in response to repeated exposure to shear stress may be related to changes in vasodilator (e.g., NO‐pathway, prostacyclin) or vasoconstrictor (e.g., ET) pathways or SNS (Green et al., 2017). Alternative pathways may involve the mobilization of endothelial progenitor cells (EPCs) from the bone marrow (Erkens et al., 2017). An increase in circulating EPC levels has been observed after (systemic) aerobic exercise (Lenk et al., 2011) and following resistance exercise (40 min at 60% 1‐RM) (Waclawovsky et al., 2016), with EPC mobilization being strongly dependent on (exercise‐induced) eNOS activation (Aicher et al., 2003; Laufs et al., 2004). Consequently, elevation of EPC levels following chronic (local) aerobic exercise may be involved in vascular regeneration and angiogenesis (Asahara et al., 1997). However, further research is needed to explore and better understand the potential underlying mechanisms explaining the adaptation in vascular function and structure following local exercise.

### 3.2 Central haemodynamics

Traditionally, it is assumed that blood pressure‐lowering effects are primarily achieved by whole‐body exercise. However, in the past decade, ample evidence showed that also local aerobic exercise training has anti‐hypertensive effects. More specifically, several meta‐analyses have now demonstrated that local isometric exercise training lowers systolic and diastolic blood pressure (Edwards et al., 2024, 2023, 2022; Inder et al., 2016; Smart et al., 2019), with the most recent meta‐analysis (18 RCTs, 628 individuals, intervention of 2–12 weeks, including handgrip, wall squat or leg extension exercises) showing decreases of 9.35 mmHg (95% CI: 7.80–10.89 mmHg) and 4.3 mmHg (95% CI: 3.01–5.60 mmHg), respectively (Edwards et al., 2022). In the largest study in this meta‐analysis including normotensive individuals (*n* = 36), Badrov et al. (2013) found a significant decrease in systolic blood pressure following 8 weeks of isometric handgrip exercise of 6 mmHg (from 94 ± 6 to 88 ± 5 mmHg, and from 97 ± 11 to 91 ± 9 mmHg) for groups exercising 3 and 5 times per week, respectively (4 × 2 min with 4 min rest, 30% MVC), while no changes were seen in the control group. A clinically relevant reduction (>2 mmHg) was observed in 75% and 78% of the participants, respectively. No reduction in diastolic blood pressure was seen, possibly due to the already low baseline blood pressure values (Badrov et al., 2013). Studies have found these blood pressure‐lowering effects already within 3 weeks, but also following relatively low levels of exercise intensity (i.e., <50% MVC) (Loaiza‐Betancur & Chulvi‐Medrano, 2020; Smart et al., 2019), which raises questions regarding potential mechanisms underlying blood pressure‐lowering effects.

Since blood pressure is determined by cardiac output and TPR, chronic changes in blood pressure are likely accompanied by changes in these factors (Edwards et al., 2024). Studies on the effects of local exercise training on heart rate show conflicting results, with some showing lower heart rates (Baross et al., 2012; Devereux et al., 2010; Wiles et al., 2017) and others no change (Baddeley‐White et al., 2019; Badrov et al., 2013; Carlson et al., 2016; Okamoto et al., 2020; Taylor et al., 2019). Similarly, local exercise training does not alter stroke volume (Edwards et al., 2022; O'Driscoll et al., 2022), making it unlikely that the effects of local exercise training on blood pressure are explained through changes in cardiac output. This makes changes in TPR a more likely contributor to the reductions in blood pressure. Indeed, a decrease in TPR concurrent with a reduction in resting systolic and diastolic blood pressure has been observed following local isometric handgrip or wall squat training (Edwards et al., 2022; O'Driscoll et al., 2022). Under resting conditions, the forearm vascular bed only minimally contributes to TPR. It is, therefore, unlikely that changes in vascular tone in the exercising muscles alone can explain the decrease in TPR, and thus blood pressure.

**FIGURE 2:**
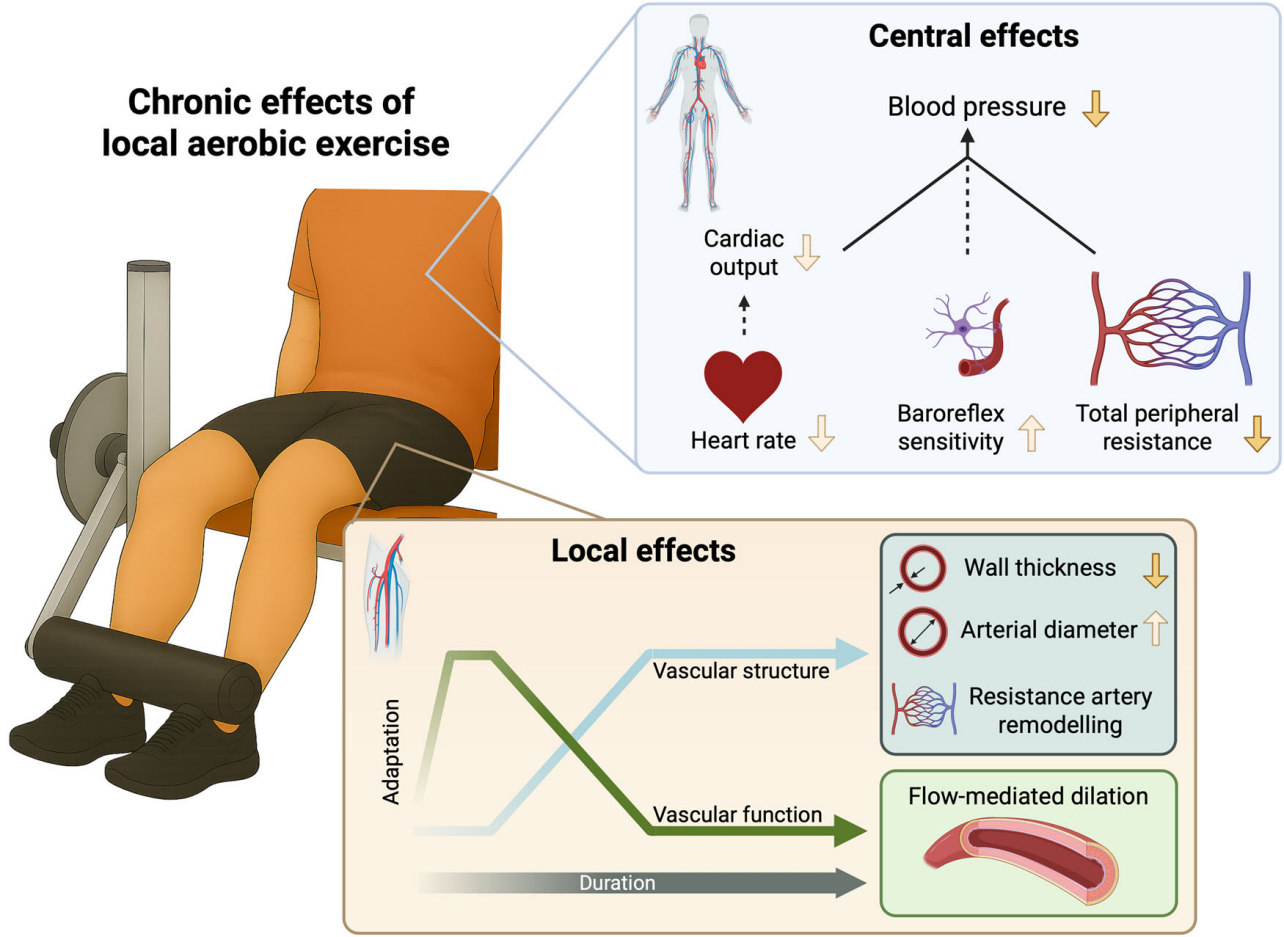
Chronic effects of local aerobic exercise, represented as central effects (adaptations in blood pressure) and local effects. For the local effects, adaptations in vascular function (flow‐mediated dilation) precede adaptations in vascular structure (wall thickness, arterial diameter and resistance artery remodelling).

Several other interconnected pathways may be involved. First, localized rhythmic contractions are known to modulate autonomic balance, shifting cardiovascular control from sympathetic to parasympathetic activity. Indeed, a previous study adopted microneurography and showed attenuated muscle sympathetic nerve activity following 6 weeks of isolated limb training (Ray & Hume, 1998). In agreement with this, improvements in baroreflex receptor sensitivity and the autonomic regulation of the heart (e.g., heart rate, contractility, vagal tone) were found following local aerobic exercise training (Edwards et al., 2022). In parallel, repeated bouts of local exercise can improve local vasomotor control through enhanced endothelial function, increased NO bioavailability, and reduced oxidative stress (see section 2.1.2.). Beyond these local vascular effects, evidence suggests that local aerobic exercise can induce systemic vascular adaptations, including reduced arterial stiffness and left ventricular afterload (Edwards et al., 2023; Okamoto et al., 2020). Nonetheless, the mechanisms underlying such adaptations remain to be elucidated. Figure 2 provides an overview of the chronic local and central effects of local aerobic exercise.

## 4 WHAT FACTORS IMPACT THE ACUTE AND CHRONIC RESPONSES TO LOCAL AEROBIC EXERCISE?

### 4.1 Subject characteristics

#### 4.1.1 Age, CVD and medication

Older age is accompanied by various changes in physiological responses to acute exercise. First, at systemic level, advancing age attenuates the absolute heart rate response to static handgrip exercise (at 30% MVC, 2 × 3 min with no rest in between (Krzemiński et al., 2012) and at 30, 40 and 50% MVC, all 4 × 20 s, with 1 min rest in between (Lalande et al., 2014)), possibly due to a decline in parasympathetic withdrawal and sympathetic activation associated with older age (Stratton et al., 2003). This results in a blunted increase in cardiac output during local aerobic exercise compared to younger individuals (Krzemiński et al., 2012; Lalande et al., 2014). Second, at a local level, higher age is associated with an attenuated exercise‐induced increase in blood flow and shear rate, which is likely due to an impaired local contraction‐induced rapid vasodilation and/or blunted functional sympatholysis (Carlson et al., 2008; Casey & Joyner, 2012; Gonzales et al., 2009). Underlying processes may include impaired production of NO and prostaglandins, due to endothelial dysfunction, as well as an increased production of endothelin‐1 (Hearon & Dinenno, 2016). An age‐related increase in oxidative stress may also play a role (Hearon & Dinenno, 2016), as NO is scavenged by excess ROS, which activates inflammatory pathways, such as NF‐κB (Harrison et al., 2006). These processes may suppress the beneficial shear‐induced adaptations to local aerobic exercise. Furthermore, with advancing age, there is hyperplasia of the intima, accumulation of collagen of the media, and accumulation of calcium and phosphate in elastin fibres (Boss & Seegmiller, 1981). These structural changes associated with ageing contribute to increases in arterial stiffness and thereby alter haemodynamic responses. Consequently, these age‐related processes may attenuate physiological response in response to exercise in older age.

Also in CVD, such as heart failure, changes in physiological responses to local aerobic exercise occur. For example, blood flow responses and vascular conductance were attenuated during handgrip (15, 30, 45% MVC at 60 contractions/min, each for 3 min with 5 min rest in between) and knee‐extensor exercises (0, 5, 10, 15 W at 60 contractions/min, each for 3 min with 5 min rest in between) in patients with heart failure (Barrett‐O'Keefe et al., 2019), especially at higher intensities (Barrett‐O'Keefe et al., 2014; Ratchford et al., 2020). This attenuation of blood flow may be due to enhanced α‐adrenergic‐mediated vasoconstriction compared to healthy participants, which may lead to impaired peripheral vasodilatory capacity and functional sympatholysis (Barrett‐O'Keefe et al., 2014, 2019). Therefore, disease‐related vascular dysregulation of the skeletal muscle resistance vasculature may also explain the attenuation of blood flow and vascular conductance, but exact disease‐related mechanisms remain to be elucidated.

These impaired haemodynamic responses to acute exercise in older and/or diseased subjects may translate to distinct patterns of adaptation of vascular function and structure compared to healthy, young individuals. Indeed, studies examining conduit artery diameter in response to local aerobic exercise training reported little to no change in patients with peripheral artery disease (isometric handgrip at 30% MVC for 4 × 2 min, with 4 min rest in between, 4 days per week for 8 weeks) (Correia et al., 2020) and hypertension (isometric exercise at 30% MVC for 4 × 2 min, alternating hands, 3 days per week for 12 weeks) (Cahu Rodrigues et al., 2020), a finding which contrasts to healthy individuals (isometric leg extensions at 14% MVC, 4 × 2 min with 2 min rest, 3 days per week for 8 weeks) (Baross et al., 2012).

Additionally, when examining local aerobic exercise training in older subjects or those with a priori CVD or risk, most studies utilizing FMD demonstrate a gradual improvement in endothelium‐dependent vasodilation. Indeed, a meta‐analysis in 179 subjects with CVD or risk (7 studies, TESTEX risk of bias range 8–13) illustrated an increase in brachial artery FMD following ≥4 weeks of isometric handgrip training of 3.32% (95% CI: 1.68–4.96) (Silva et al., 2021). These are clinically meaningful improvements as already 1% change in FMD relates to an 8–13% risk reduction for CVD (Inaba et al., 2010; Ras et al., 2013; Thijssen et al., 2019). For example, McGowan, Visocchi et al. (2007) showed a 4.2% increase in local FMD following 8 weeks of unilateral handgrip exercise (4 × 2 min with 4 min in between, 30% MVC for 3 days per week) in medicated hypertensive participants. Nonetheless, this observation contrasts with healthy individuals, who show no change in FMD across prolonged training with a similar intensity, dose and frequency (McGowan, Levy et al., 2007) (see section 3.1). This divergence may be explained by differences between groups. In populations with endothelial dysfunction, such as those with hypertension or early atherosclerotic changes, baseline NO‐mediated vasodilation is impaired, oxidative stress is elevated and shear stress‐dependent signalling pathways are blunted (Harrison et al., 2006). Local aerobic exercise provides a potent, repeated shear stress stimulus that upregulates endothelial NO synthase, enhances NO bioavailability and reduces ROS, thereby restoring vasomotor function (Harrison et al., 2006). The relative capacity for improvement in these individuals, therefore, is larger than in healthy, young individuals.

Nonetheless, distinct adaptations have been observed when examining PWV in various clinical populations. In contrast to the improvement in arterial stiffness found in healthy individuals (Edwards et al., 2023; Okamoto et al., 2020), no change in PWV or augmentation index was found after 8 and 12 weeks of isometric local aerobic exercise training in patients with peripheral artery disease (isometric handgrip at 30% MVC for 4 × 2 min, with 4 min rest in between, 4 days per week for 8 weeks) (Correia et al., 2020) or hypertension (isometric handgrip exercise at 30% MVC for 4 × 2 min with 1 min rest, 3 days per week for 12 weeks) (Farah et al., 2018). One potential reason may relate to vascular abnormalities and the presence of higher arterial stiffness in patients with peripheral artery disease (Edwards et al., 2024). As PWV is strongly affected by structural characteristics of the arterial wall, changes in PWV may be relatively difficult to achieve (Zieman et al., 2005). In line with this, the duration of the intervention may also be a limiting factor for rapid systemic changes to occur (Edwards et al., 2024). Literature on this topic is limited, and more longitudinal data (>12 weeks) are warranted to unravel the exact physiological mechanisms of systemic structural vascular remodelling and/or functional changes underlying changes in arterial stiffness in distinct populations.

With respect to blood pressure, a meta‐analysis incorporating 302 participants demonstrated larger reductions in mean blood pressure for older (≥45 years) than younger (< 45 years) subjects, following 3–10 weeks of regular local aerobic exercise (−5.51 vs. −2.72 mmHg, respectively) (Inder et al., 2016). Similarly, participants with hypertension demonstrated a larger reduction in mean blood pressure than normotensive subjects (−5.91 vs. −3.01 mmHg, respectively) (Inder et al., 2016). These larger effects in older and hypertensive individuals are likely due to their higher baseline blood pressure, implicating a greater potential for improvement (Inder et al., 2016). However, not all studies demonstrate differences between normo‐ and hypertensive individuals (Edwards et al., 2022). Some of these conflicting observations may be related to the use of anti‐hypertensive medication. For example, changes in inflammatory biomarkers (e.g., interleukin‐6) have been reported following local aerobic exercise training in untreated hypertensive individuals (Javidi et al., 2022; Taylor et al., 2019), while this was not found in medicated hypertensive subjects (Rodrigues et al., 2020). Since training and anti‐hypertensive medication report overlapping mechanisms to lower blood pressure, the use of medication may attenuate the potential for exercise training to further lower blood pressure (Edwards et al., 2024). The large number of antihypertensive drugs, each with different mechanistic effects, limits our current understanding of the exact role of anti‐hypertensive medication to explain the conflicting results (Edwards et al., 2024).

#### 4.1.2 Biological sex

Relevant biological sex‐based differences are present regarding vascular health. Compared to males, females have smaller conduit artery diameters as well as higher compliance and lower arterial stiffness (Fekete et al., 2025). Previous work found that the menstrual cycle affects arterial compliance, which suggests a role for oestrogen to mediate these differences in vascular function and structure. Moreover, vascular compliance decreases after menopause, further supporting this hypothesis (DuPont et al., 2019). Similarly, fluctuations in oestrogen across the menstrual cycle may affect FMD (Thijssen et al., 2019), whilst premenopausal women present higher NO bioavailability, most likely via upregulation of eNOS through oestrogen, which is associated with higher vascular function and lower levels of inflammation (Lv et al., 2025). These observations support the presence of sex‐based differences in vascular function and structure. However, literature on the effect of biological sex on response to local aerobic exercise training is limited. Regarding vascular responses, Gonzales et al. (2009) found that during knee extensor exercise (40 contractions/min, incrementally increasing resistance), women showed a larger increase in shear rate response than men, which persisted as the work increased. Oliveira et al. (2021) found no differences in the change of brachial artery diameter and FMD between older men and women following 8 weeks of isometric handgrip training (30% MVC, 4 × 2 min with 4 min rest in between, 3 days per week). Finally, a meta‐analysis reported no differences regarding blood pressure reductions between males and females (Inder et al., 2016). Taken together, although research in this field is limited and despite the presence of sex‐based differences in vascular structure and function, there is currently no support for presence of sex differences in response to acute and chronic local aerobic exercise training.

### 4.2 Exercise characteristics

Responses to local aerobic exercise training may be affected by exercise characteristics, including intensity, training volume (duration and frequency) and muscle volume. For example, a dose–response relation is strongly present between the intensity of the exercise and acute vascular responses (Baross et al., 2012; Carlson et al., 2016). Higher exercise intensity amplifies endothelial NO‐dependent signalling and favours functional improvements such as enhanced FMD and vasodilator responsiveness (Baross et al., 2012; Harrison et al., 2006). When high intensity is sustained over weeks, the repeated elevation in peak shear and intraluminal pressure can also promote outward remodelling (increased lumen diameter) of the supplying artery, especially when combined with sufficient volume (Baross et al., 2012). Even at moderate intensities, higher weekly volume (longer sessions and/or more frequent training) accumulates repeated episodes of elevated flow and wall stress in the arteries supplying the trained muscle (Baross et al., 2012). Regarding the muscle volume, previous work found no differences in improvement in remote brachial artery vascular function (FMD) between handgrip (50% MVC, 25 reps/min for 4 × 5 min, 5 min rest in between) or squat exercises (15 squats/min for 4 × 5 min, 5 min rest in between) (Somani et al., 2024). However, it is important to consider that comparing small (i.e., upper limbs) and larger (i.e., lower limbs) muscle mass is challenging since intensities of both types of exercise differ substantially. As the intensity of exercise affects outcomes, this hampers comparisons between limbs or muscle groups.

Beyond traditional descriptors of exercise dose, the contraction mechanics and duty cycle (ratio of contraction to relaxation time) also impact local haemodynamics (Broxterman et al., 2014). Experimental work shows that identical external work rates can produce markedly different oxygen uptake and blood flow responses when contraction frequency or duty cycle is manipulated (Broxterman et al., 2014). For example, a relatively long muscle contraction cycle limits blood flow and oxygen uptake (Broxterman et al., 2014). The effects of differing contraction mechanics and duty cycle on endothelial function remain to be elucidated.

**FIGURE 3:**
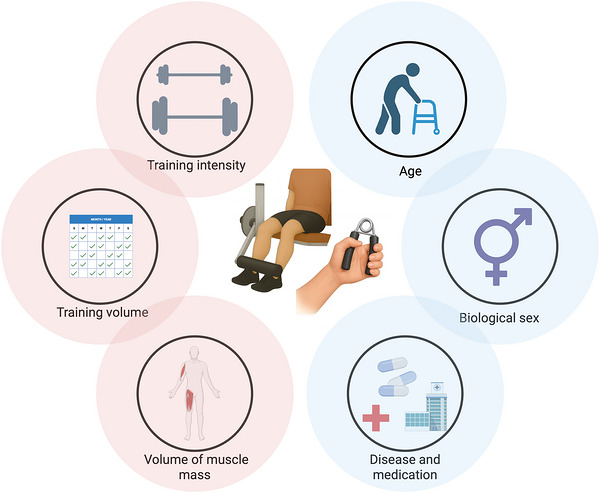
Exercise and subject characteristics that may affect responses of chronic exposure to local aerobic exercise. Characteristics highlighted in red represent the exercise characteristics, while the characteristics highlighted in blue represent the subject characteristics in response to chronic exposure of local aerobic exercise (e.g., leg extension or handgrip training).

In line with local vascular adaptations, a clear dose–response relation is also observed for systemic blood pressure in relation to exercise intensity. For example, Baross et al. (2012) compared 8 weeks of isometric leg exercises performed at 8% versus 14% MVC (4 × 2 min with 2 min rest, for 3 days per week) and found significant reductions in resting blood pressure in the group undergoing exercise at the highest exercise intensity. Similarly, Carlson et al. (2016) found a larger effect on systolic blood pressure following 8 weeks of isometric handgrip exercises at 30% MVC (−7 mmHg) compared to 5% MVC (−4 mmHg). Additionally, a dose–response is found in the relation between the duration and volume of exercise and changes in blood pressure. More specifically, a meta‐analysis reported that ≥8 weeks of isometric local aerobic exercise training resulted in a larger reduction in systolic blood pressure than <8 weeks of training (−8.47 vs. −2.99 mmHg, respectively), as well as for mean blood pressure (−4.22 vs. −1.85 mmHg) (Inder et al., 2016). After 4 weeks, Badrov *et al.* showed systolic blood pressure reductions when subjects performed 5 times, but not the 3 times isometric handgrip training per week over an 8‐week intervention (30% MVC, 4 × 2 min with 4 min rest) (Badrov et al., 2013). However, at 8 weeks, the systolic blood pressure reductions were comparable between the groups, indicating accelerated adaptations following higher training frequency and a possible plateau phase (Badrov et al., 2013). Regarding the role of volume of muscle mass in relation to blood pressure‐lowering effects, the literature is inconclusive (Edwards et al., 2022; Inder et al., 2016; Somani et al., 2024). Taken together, intensity and training volume, but unlikely the volume of muscle mass, demonstrate a dose‐dependent effect for both local and central adaptations to local aerobic exercise training (Figure 3).

## 5 CONCLUSION AND FUTURE PERSPECTIVE

This review summarised the acute and chronic physiological effects of local aerobic exercise on the cardiovascular system. First, local aerobic exercise leads to various vascular responses, including an acute intensity‐dependent increase in blood flow, hampered by mechanical compression and driven by peripheral vasodilation. The subsequent elevation in shear stress seems primarily responsible for immediate improvements in vascular function that return to baseline after chronic exercise training due to structural vascular remodelling. In addition to these potentially cardioprotective effects, studies report that local aerobic exercise may also induce local and remote protection against endothelial IR injury, either acutely or chronically. Furthermore, despite activating a relatively small muscle mass, blood pressure rises during local aerobic exercise. More importantly, local aerobic exercise leads to a clinically relevant reduction in resting blood pressure when performed regularly. Finally, it is important to highlight that several factors affect these acute and chronic physiological responses, including subject‐related (i.e., age, disease and medication status) and exercise‐related (i.e., intensity, training volume) factors, whereas the effects of biological sex and muscle volume remain to be elucidated. An overview of all remaining research gaps providing inspiration for future studies is displayed in Table 1. Since local aerobic exercise is relatively simple, low‐cost and widely accessible, the cardiovascular adaptations may offer clinically meaningful benefits. This warrants further clinical investigation – for example, as a prehabilitation strategy for patients awaiting cardiac surgery.

**TABLE 1:** Research gaps in the effects of local aerobic exercise on cardiovascular physiology.

Section	Research gaps
2.1.1 Blood flow and vasodilation	The relative contribution of metabolites (e.g., adenosine, K^+^, eNOS, heat shock proteins) and endothelial factors (e.g., NO, prostacyclin, EDHF) and their interplay, during local aerobic exercise
	The influence of different intensities and durations of local aerobic exercise on the metabolites and endothelial factors
2.1.2 Shear stress	Clarifying how different patterns of shear rate during local aerobic exercise affect changes in vascular function
	Clarifying how mechanical signals detecting shear stress interact with both local (endothelial, metabolic) and systemic (neural, hormonal, haemodynamic) regulatory pathways
2.2 Vascular function	Understanding how local aerobic exercise alters vascular function (e.g., endothelial responsiveness, shear‐mediated signalling and microvascular regulation) and the role of exercise characteristics herein
	Understanding how acute responses to exercise relate to or contribute to long‐term vascular adaptations
2.3 Endothelial ischaemia–reperfusion injury	Understanding signalling pathways through which local aerobic exercise confers protection against ischaemia–reperfusion injury, and how these mechanisms differ from other preconditioning stimuli
	Clarifying how individual characteristics (e.g., age, cardiovascular risk profile) modify protective responses of exercise on endothelial ischaemia–reperfusion
2.4 Central haemodynamics	Understanding how central haemodynamic adjustments during local aerobic exercise interact with the local vascular effects
3.1 Vascular adaptations	Understanding the mechanisms that drive structural vascular remodelling after long‐term local aerobic exercise
3.2 Central haemodynamics	Developing a clearer understanding of how local aerobic exercise induces systemic adaptations (reductions in blood pressure and TPR)
	Clarifying underlying mechanisms linking localized vascular stimuli (e.g., shear stress, metabolic signalling) to whole‐body haemodynamic/autonomic changes
4.1.1 Age, CVD and medication	Understanding how CVD interacts with exercise hyperaemia during local aerobic exercise, and how this affects the acute and long‐term vascular responses
	Understand how medication influences acute vascular responses to exercise, but also interacts with training‐induced vascular remodelling and vascular function
4.1.2 Biological sex	Understanding sex‐related differences in both the acute vascular responses and the chronic adaptations to local aerobic exercise training
	Clarifying how biological sex influences the underlying endothelial, autonomic and structural mechanisms driving the acute and chronic responses
4.2 Exercise characteristics	Understanding how the amount of active muscle mass affects acute (e.g., blood flow and shear stress) and longer‐term structural and functional cardiovascular adaptations

## AUTHOR CONTRIBUTIONS

Myrthe van de Ven: Conceptualization, writing—original draft. Janneke Vloet: Visualization, writing—review and editing. Daphne Merkus: Writing—review and editing. Dick Thijssen: Conceptualization, supervision, writing—review and editing. All authors approved the final version of the manuscript and agree to be accountable for all aspects of the work in ensuring that questions related to the accuracy or integrity of any part of the work are appropriately investigated and resolved. All persons designated as authors qualify for authorship, and all those who qualify for authorship are listed.

## CONFLICT OF INTEREST

None declared.

## GENERATIVE AI STATEMENT

Generative AI was used for the production of the graphical element ‘hand with the handgrip device’ in Figure 1 and 3 and the ‘leg press’ in Figure 2 and 3 (ChatGPT).
